# Septin-dependent invasive growth by the rice blast fungus *Magnaporthe oryzae*

**DOI:** 10.1007/s41348-024-00883-4

**Published:** 2024-03-07

**Authors:** Iris Eisermann, Nicholas J. Talbot

**Affiliations:** The Sainsbury Laboratory, University of East Anglia, Norwich Research Park, Norwich, NR47UH UK

**Keywords:** Septins, Appressorium, Invasive growth, Plant pathogens, Fungi, Fungicide

## Abstract

Septin GTPases are morphogenetic proteins that are widely conserved in eukaryotic organisms fulfilling diverse roles in cell division, differentiation and development. In the filamentous fungal pathogen *Magnaporthe oryzae*, the causal agent of the devastating blast diseases of rice and wheat, septins have been shown to be essential for plant infection. The blast fungus elaborates a specialised infection structure called an appressorium with which it mechanically ruptures the plant cuticle. Septin aggregation and generation of a hetero-oligomeric ring structure at the base of the infection cell is indispensable for plant infection. Furthermore, once the fungus enters host tissue it develops another infection structure, the transpressorium, enabling it to move between living host plant cells, which also requires septins for its function. Specific inhibition of septin aggregation—either genetically or with chemical inhibitors—prevents plant infection. Significantly, by screening for inhibitors of septin aggregation, broad spectrum anti-fungal compounds have been identified that prevent rice blast and a number of other cereal diseases in field trials. We review the recent advances in our understanding of septin biology and their potential as targets for crop disease control.

## Introduction

The rice blast fungus *Magnaporthe oryzae* (synonym of *Pyricularia oryzae*) causes the most devastating disease of rice worldwide (Zhang et al. 2016; Wang et al. 2009), with losses estimated at $66 billion (Pennisi 2010). In the USA alone—a country responsible for only 1–2% of global rice production—fungicides worth $70 million are needed each year to control rice blast disease (Nalley et al. 2016; Cruz and Valent 2017). However, an even greater threat to the global food security is presented by the entire *M. oryzae* disease complex, because different host-specific forms of *M. oryzae* can cause blast disease on millets, barley, oats and wheat (Gladieux et al. 2018; Valent et al. 2019; Kato et al. 1977). Outbreaks of finger millet blast affect smallholder farmers in southern Asia and eastern Africa, causing hardship to resource-poor communities (Takan et al. 2012). Wheat blast outbreaks were until recently restricted to South America, but since 2016 have spread into Bangladesh and from 2020 into Zambia, potentially threatening major wheat-producing regions in South Asia and Africa (Singh et al. 2021, Mbinda et al. 2021, Latorre et al. 2023).

Rice-infecting strains of *M. oryzae* cause disease symptoms on leaves, necks, and panicles of rice plants (Valent et al. 1991). Disease is initiated by development of a dome-shaped infection structure called the appressorium, which generates turgor that is exerted onto the leaf surface to rupture the rice cuticle. The fungus sends a rigid penetration hypha into the epidermis and this further develops into specialised intracellular invasive hyphae, which enter and feed in living host cells (Valent 2021; Cruz-Mireles et al. 2021a, b; Ryder et al. 2022). The fungus moves to new rice cells via pit field sites where plasmodesmata accumulate, using a specialised structure termed a transpressorium (Cruz-Mireles et al. 2021a, b). The hyphal tip swells and then undergoes severe hyphal constriction to enable the fungus to move into new cells, whilst maintaining rice cellular integrity (Sakulkoo et al. 2018). To facilitate infection, the fungus also secretes a battery of effector proteins that suppress plant immunity and enable rapid proliferation of fungal hyphae in living plant tissue (Yan et al. 2023). The fungus then develops aerial conidiophores from the centre of necrotic disease lesions, which produce conidia to infect new plants (Ryder et al. 2022). The precise developmental transitions involved in the biology of plant infection by *M. oryzae* suggest key roles for septins both during the early stages of infection and invasive growth.

Septins are guanosine triphosphatases (GTPases) that function in many distinct cellular processes such as polarity determination, secretion, cytokinesis and endocytosis. Septins were first reported in 1971 in *Saccharomyces cerevisiae*, where it was shown that septins play a role during cell division, forming a ring at the bud site prior to budding of a new yeast cell. Like actin and microtubules, septins form a further component of the cytoskeleton and are expressed in all eukaryotes except plants (Van Ngo et al. 2019; Hartwell et al. 1974; Spiliotis et al. 2006; Douglas et al. 2005). Septins possess a specific domain structure. The central GTP-binding domain is flanked by N- and C-terminal regions. A polybasic region (PBR) is responsible for interaction with the membrane as it binds to negatively charged phospholipids and is located between the N-terminal region and the GTP-binding domain. A septin unique element (SUE) downstream of the GTP-binding domain is proposed to be involved in septin polymerisation and is located before the C-terminal region. The C-terminal region itself is involved in septin-protein interactions (Van Ngo et al. 2019; Sirajuddin et al. 2009; Versele et al. 2004).

**Fig. 1:**
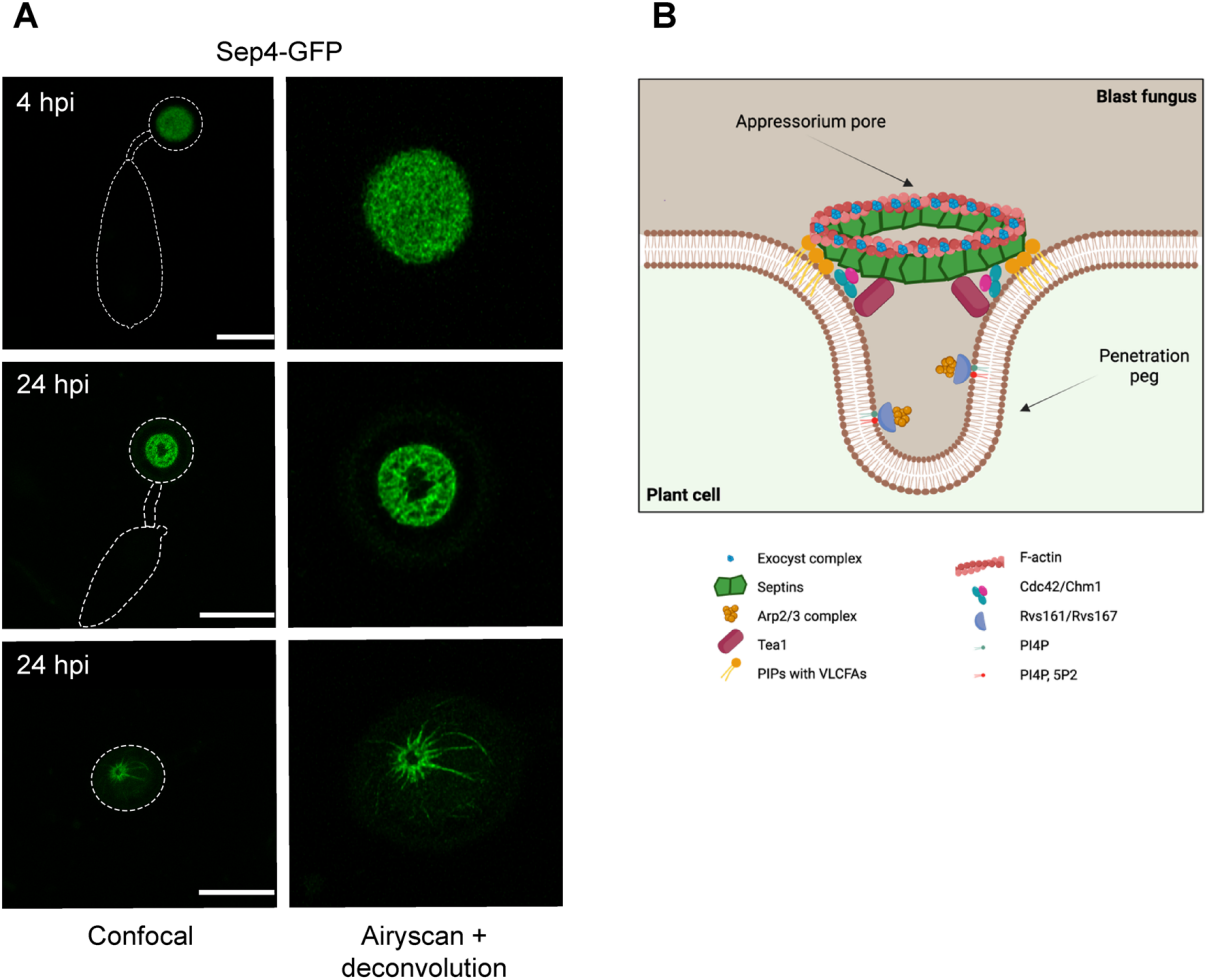
Core septins oligomerise and assemble into a disc-like structure that subsequently contracts, shaping into a ring around the appressorium pore. **A** Cellular localisation of Sep4-GFP at the appressorium pore. Visualisation by laser scanning confocal microscopy and processed with Airyscan joint deconvolution at 4 and 24 h post inoculation. The Sep4-containing septin disc reaches its biggest volume at 4 h to then contract until it starts forming a ring from which then later rays emanate. The later structure is especially observed on plant surfaces. Scale bar indicate 5 μm. **B** Scheme of the septin ring formation at the appressorium pore with known septin-interacting proteins at the point of rice cuticle penetration

Septins assemble into dynamic hetero-oligomeric complexes and form higher-order structures such as bars, gauzes, rods, discs, and rings by interacting with themselves and with other septins (Sirajuddin et al. 2007; Dagdas et al. 2012; Bridges and Gladelter 2015). *M. oryzae* possesses six septins of which the four septin genes *SEP3*, *SEP4*, *SEP5* and *SEP6* are homologs of the *S. cerevisiae* core spetin genes *CDC3*, *CDC10*, *CDC11* and *CDC12* which are involved in cytokinesis (Dagdas et al. 2012). The two non-core septins *SEP7* and *SEP8* belong to the class 5 group of septins, which is absent in humans and yeasts and found predominantly in filamentous fungi (Shuman and Momany 2022; Eisermann et al. 2023). During the early stages of infection, the four core septins Sep3, Sep4, Sep5 and Sep6 form a large hetero-oligomeric disc- and later a ring-structure at the base of the appressorium surrounding the penetration pore (see Fig. 1A) (Dagdas et al. 2012; Dulal et al. 2020).

## The function of septins during the early stages of plant infection

Septins have been implicated in appressorium function, serving a vital role in the ability of these cells to rupture the host plant cuticle. The dome-shaped, melanin-pigmented appressorium attaches tightly to the leaf surface and expands due to an increase in turgor pressure (Wilson and Talbot 2009; Ryder et al. 2019). Appressorium development is regulated by activation of the Pmk1 MAP kinase pathway (Xu and Hamer 1996). Recognition of surface signals and Pmk1 phosphorylation leads to large changes in phosphorylation of the *M. oryzae* proteome (Cruz-Mireles et al. 2023), and a hierarchical transcriptional network is then activated to control appressorium morphogenesis. The conidium, from which the appressorium develops, undergoes autophagic cell death, which is also a Pmk1-dependent process and is necessary for transfer of the contents of the three-celled spore to the appressorium to allow generation of the enormous turgor pressure of up to 8.0 MPa (Veneault-Fourrey et al. 2006; Osés-Ruiz et al. 2021). The process of appressorium formation is also tightly linked to cell cycle progression. An S-phase checkpoint is necessary for appressorium development (Saunders et al. 2010) and a second checkpoint is required for maturation of the appressorium (Osés-Ruiz et al. 2017).

As the appressorium matures and becomes melanised, the four core septins Sep3, Sep4, Sep5 and Sep6 form a large hetero-oligomeric disc at the appressorium base, which then develops into a ring structure at the appressorium pore, surrounding the point of penetration peg emergence (see Fig. 1A). This acts to recruit a F-actin network to the appressorium base, re-modelling the cytoskeleton to facilitate re-polarisation of the infection cell (Dulal et al. 2020; Dagdas et al. 2012; Eisermann et al. 2023). Both, septin and F-actin ring assembly requires the regulated generation of ROS via an NADPH oxidase complex (Egan et al. 2007; Ryder et al. 2013). Septin assembly reqires the Sln1-dependent turgor sensing pathway, which monitors sufficient turgor generation as well as progression of the appressorial nucleus through the S-phase (Ryder et al. 2019; Osés-Ruiz et al. 2017). Sln1 regulates septin ring formation by activating the Nox2-NoxR NADPH oxidase complex via the Pkc1-dependent cell-integrity pathway, which leads to initiation of polarised growth of the penetration peg, where Nox1 is then required for the elongation of the penetration peg and Nox2/NoxR are necessary for the peg to be formed (Ryder et al. 2013). Septin ring assembly is a complex process involving many components (see Fig. 1B). The small Rho GTPase Cdc42, for example, is one of those components and acts as a polarity determinant required for septin ring assembly. In addition the p21-activated kinase Chm1 (an ortholog of Cla4) is likely to phosphorylate septins at the appressorium pore (Dagdas et al. 2012). It is also an important regulator of conidiogenesis (Chen et al. 2008). In addition, the Ras GTPase activating protein Smo1 is indispensable for septin recruitment. Smo1 negatively regulates the Ras2 signalling complex and interacts with the autophagy proteins Atg3, Atg4, Atg5 and Atg7, but also directly with the four core septins and components of the exocyst complex (Kershaw et al. 2019). Smo1 mutants are non-pathogenic, affected in appressorium morphogenesis and also produce aberrant round, two celled-conidia (Kershaw et al. 2019). Collectively, these components are essential to enable septin organisation and re-modelling of the F-actin network at the appressorial base by building phosphoinositide linkages with the plasma membrane and facilitating F-actin plasma membrane linkages via ezrin-radixin-moesin (ERM) proteins, such as Tea1, which is also regulated by the cyclic AMP (cAMP) protein kinase A (PKA) and Pmk1 MAPK pathways during appressorium formation (Qu et al. 2022; Dagdas et al. 2012). In this context, deletion of the F-actin cross-linker fimbrin, affects polarity of the actin cytoskeleton and disrupts F-actin ring aformation at the base of the appressorium which also affects assembly of the exocyst (Zhang et al. 2022; Li et al. 2020). Microtubules also become arranged in a perpendicular way to the F-actin network, which is also a septin-dependent process (Dulal et al. 2020).

In addition to its role in scaffolding and re-organising F-actin, the septin ring also functions as a lateral diffusion barrier for correct positioning of specific actin-associated proteins, such as Las17 which polymerises F-actin via the Arp2/3 complex, gelsolin an actin-severing protein necessary for correct dynamic assembly of F-actin, coronin which promotes F-actin remodelling, and Rvs167, associated with endocytosis (see Fig. 1B) (Dagdas et al. 2012; Dulal et al. 2021). Interestingly the endocytic protein Pal1, which functions upstream of the cAMP and the Pmk1 MAPK pathway, also influences the distribution of Sep5 and Sep6 at the base of the appressorium (Chen et al. 2022). The septin lateral diffusion barrier also secures localisation of the octameric exocyst complex to the appressorium pore, which is necessary for polarised exocytosis and the emergence of the penetration peg. Co-immunoprecipitation experiments have revealed an interaction between the exocyst proteins Sec6 and Exo84 with all four core septins, as well as with Rho1 and Rac1 (Rho GTPases), fimbrin, and Pmk1 (Gupta et al. 2015). Interestingly, it has recently been shown that some effector proteins, implicated in suppression of plant immunity, are expressed prior to plant infection and may be secreted at the base of the appressorium prior to penetration peg development which is likely to be septin-dependent (Yan et al. 2023).

## Determinnig the function of septins during invasive growth of the blast fungus

Once *M. oryzae* has ruptured the rice cuticle and is growing within the first epidermal rice cell, the penetration hypha differentiates into specialised invasive hypha and the fungus undergoes major changes in its primary metabolism, switching to growth that is dependent on sequestration of nutrients from the host rather than storage products in the spore (Fernandez et al. 2014). A plant membrane-rich cap called the biotrophic interfacial complex (BIC) develops at the tip of the penetration hypha and remains in place as the invasive hypha undergoes pseudohyphal growth. An emerging body of evidence suggests that the BIC is likely to be the site of effector secretion and delivery into plant cells (Kankanala et al. 2007; Khang et al. 2010). Recent evidence has, for example, shown the BIC to be the site of clathrin-mediated endocytosis of effector-containing membranous bodies into rice cells (Oliveira-Garcia et al. 2023). *M. oryzae* differentiates into multiple bulbous, branched hyphae, which fill the epidermal cell almost completely, and then invade neighbouring cells by crossing through pit fields. As the fungus moves to the next living neighbour plant cells, the initially colonised plant cells lose viability (Valent 2021; Oliveira-Garcia et al. 2023). Live-cell imaging has revealed that hyphal tips swell and can reach a diameter of ~ 5.0 µm, before constricting to 0.3–0.8 µm in diameter as they pass through pit fields into adjacent cells (Sakulkoo et al. 2018).

Because of the similarity in development of the appressorium, Liese and Schmid were the first in 1964 to name fungal cell-to-cell invasive structures “transpressoria”. It now seems likely that appressorium and transpressorium development are morphogenetically-related because formation of both structures involves initial isotropic expansion of a swollen germ tube tip, followed by re-polarisation and formation of a much narrower penetration peg, or penetration hypha, respectively (Liese et al. 1964; Cruz-Mireles et al. 2021a, b). However, the transpressorium is not melanised in the same way as the appressorium, as the turgor required for cell-to-cell movement is unlikely to be as great as that necessary for cuticle rupture. However, it will be interesting in future to apply new methods that enable membrane tension to be directly measured (Ryder et al. 2023) to see if turgor does play a role at the pit field sites in enabling fungal invasion, in the same way as observed in appressoria.

Interestingly, transpressorium-mediated penetration in *M. oryzae* also appears to be septin-mediated because septin rings localise at the rice cell crossing points (Sakulkoo et al. 2018). Moreover, septin-deficient mutants are impaired in their ability to carry out invasive growth in the rare instances when invasive hyphae are formed (Sakulkoo et al. 2018). By using an analogue-sensitive mutant of Pmk1, inhibition of the MAPK pathway was also shown to prevent cell-to-cell movement in *M. oryzae*, and *pmk1*^*AS*^ mutants become trapped within infected plant cells when the MAPK is inhibited. Given the role of the transpressorium in penetrating a structural barrier in the same way as an appressorium, it is likely that many septin-dependent components are conserved in both processes (Sakulkoo et al. 2018). How septins aggregate at hyphal tips that encounter pit fields, however, is not known. Septins have been shown to sense membrane curvature and interact with other proteins, such as BAR domain protein, which are capable of sensing even lower membrane curvatures (Bridges et al. 2016). It is possible that a specific change in fungal membrane curvature could occur when invasive hyphae undertake cortical scanning and locate a pit field indentation, perhaps leading to septin aggregation and assembly of the re-polarisation apparatus. Furthermore, it was shown that the organisation of septins in *M.oryzae* appressoria requires synthesis of very long chain fatty acids (VLCFAs) that may act as mediators of septin interactions at membrane interfaces, a process that could also be important for cell-to-cell movement (He et al. 2020). Transpressorium function also requires septin-mediated remodelling of actin and perhaps also remodelling of microtubule organisation (Sakulkoo et al. 2018). Septins may also provide cortical rigidification at the site of cell-to-cell movement and act as a diffusion barrier to deploy polarity and virulence determinants at the correct position for invasion of new host cells. The parallels between appressorium and transporessorium morphogenesis are therefore striking and the role of septins is pivotal in both developmental processes.

## Septins as a target for antifungal plant protection strategies

As septins are absent in plants but play a crucial role in plant infection by a plant pathogenic fungus, septins could provide an ideal target for antifungal plant protection approaches. Specific inhibitors blocking septin assembly might provide an effective means to prevent host penetration and disease.

Evidence in support of the potential of septins as fungicide targets was provided in a study that showed how inhibition of very-long-chain fatty acid (VLCFA) biosynthesis can prevent rice blast disease (He et al. 2020). It was shown that the specific VLCFA biosynthesis inhibitors metazachlor, cafenstrole and diallate were able to inhibit septin aggregation at the appressorium pore, preventing rice leaf infection by *M. oryzae*. This was validated in a field trial experiment showing highly effective rice blast disease control. Importantly, the inhibition of VLCFA biosynthesis not only prevents rice blast disease, but also infection by other fungal pathogens such as *Bipolaris maydis* (which causes southern-corn leaf blight disease of maize), *Blumeria graminis* (which causes wheat powdery mildew disease) and *Metarhizium acridum* (an entomopathogenic fungus that infects locusts), suggesting that broad spectrum fungicides targeting septin aggregation in fungal pathogens could be developed (He et al. 2020). The inhibitors tested to date, metazachlor, cafenstrole and diallate, are all herbicides and so unlikely to be effective candidates, but they provide a proof of concept of the approach and the likely value in screening for septin aggregation inhibitors. Another compound that inhibits septin ring formation in *M. oryzae*, when applied to appressoria, is melatonin. Melatonin binds to the Mps1 MAPK and inhibits its phosphorylation, which was shown to inhibit plant infection by 13 plant pathogens including fungi and oomycetes. If systematic modifications of melatonin could be made so that it would not have an effect on humans, it might provide a low-cost alternative to combat plant pathogens (Li et al. 2023). Furthermore, chitosan application to appressoria also prevents septin aggregation and re-polarisation of the appressorium by affecting NADPH oxidase-dependent synthesis of ROS (Lopez-Moya et al. 2021). Chitosan application might therefore also provide a potential antifungal compound as it not only inhibits fungal penetration of the leaf, but also improves physiological properties of the plant and is completely biodegradable (Lopez-Moya et al. 2021; Malerba and Cerana 2016; Sharif et al. 2018).

In summary, septins are important for fungal pathogenesis serving specific roles associated with the cell shape changes that occur during plant infection. In *M. oryzae,* septin aggregation in appressoria and transpressoria is essential for their function. Inhibition of septin aggregation genetically or using VLCFA biosynthesis inhibitors can, furthermore, prevent blat disease and a range of other important crop diseases, as well as insect infection by entomopathogenic fungi. The broad spectrum nature of this control provides further evidence of the pivotal role of septins in fungal developmental biology and their potential as a target for development of first-in-class new anti-fungal compounds.
